# Optical to Planar X-ray Mouse Image Mapping in Preclinical Nuclear Medicine Using Conditional Adversarial Networks

**DOI:** 10.3390/jimaging7120262

**Published:** 2021-12-03

**Authors:** Eleftherios Fysikopoulos, Maritina Rouchota, Vasilis Eleftheriadis, Christina-Anna Gatsiou, Irinaios Pilatis, Sophia Sarpaki, George Loudos, Spiros Kostopoulos, Dimitrios Glotsos

**Affiliations:** 1 Biomedical Engineering Department, University of West Attica, 12210 Athens, Greece; mrouchota@bioemtech.com (M.R.); skostopoulos@uniwa.gr (S.K.); dimglo@uniwa.gr (D.G.); 2 BIOEMTECH, Lefkippos Attica Technology Park, N.C.S.R. Democritos, 15343 Athens, Greece; vasilis.eleftheriadis@bioemtech.com (V.E.); cgatsiou@bioemtech.com (C.-A.G.); ipilatis@bioemtech.com (I.P.); ssarpaki@bioemtech.com (S.S.); george@bioemtech.com (G.L.)

**Keywords:** molecular preclinical imaging, image-to-image translation, PET, SPECT, deep learning, pix2pix, cGAN, X-ray

## Abstract

In the current work, a pix2pix conditional generative adversarial network has been evaluated as a potential solution for generating adequately accurate synthesized morphological X-ray images by translating standard photographic images of mice. Such an approach will benefit 2D functional molecular imaging techniques, such as planar radioisotope and/or fluorescence/bioluminescence imaging, by providing high-resolution information for anatomical mapping, but not for diagnosis, using conventional photographic sensors. Planar functional imaging offers an efficient alternative to biodistribution ex vivo studies and/or 3D high-end molecular imaging systems since it can be effectively used to track new tracers and study the accumulation from zero point in time post-injection. The superimposition of functional information with an artificially produced X-ray image may enhance overall image information in such systems without added complexity and cost. The network has been trained in 700 input (photography)/ground truth (X-ray) paired mouse images and evaluated using a test dataset composed of 80 photographic images and 80 ground truth X-ray images. Performance metrics such as peak signal-to-noise ratio (PSNR), structural similarity index measure (SSIM) and Fréchet inception distance (FID) were used to quantitatively evaluate the proposed approach in the acquired dataset.

## 1. Introduction

Drug discovery aims in identifying novel candidate treatments against diseases. It is a lengthy (it takes about 15 years) and costly (about 1–1.5 billion euros) process involving a laborious series of different stages, including preclinical studies [1,2].

A crucial part in the preclinical evaluation of drug discovery is performed on the basis of biodistribution ex vivo studies that aim in monitoring the interaction of radioisotope- or fluorescence-labeled candidate drugs on animal subjects invasively. However, this invasive method, although a gold standard, raises economical and ethical issues due to the large number of required animals [3]. Non-invasive methods, such as molecular imaging, have been proposed, aiming in improving animal handling and catalyzing the necessary time required for biodistribution ex vivo studies. Such methods include imaging using Positron Emission Tomography (PET), Single Photon Emission Tomography (SPECT), Computed Tomography (CT) and Magnetic Resonance Imaging (MRI), similar to those used in clinical practice. It is well proven that small animal imaging speeds up the whole process, increases accuracy and decreases cost [4,5,6].

Planar molecular imaging is another alternative since it can effectively be used to track a new tracer and study the accumulation from zero point in time post-injection. Two-dimensional fluorescence/bioluminescence optical imaging is a well-established method for this purpose [7,8], offering high sensitivity, simplicity and decreased cost while having low depth penetration, limited clinical translation and being nonquantitative [1,2]. On the other hand, both SPECT and PET radioisotopes planar imaging provide quantitative information of high spatial resolution while also retaining simplicity, low cost and high throughput properties at the expense of access to radioactivity [9,10]. In order to provide simultaneous anatomic information, both techniques have been used, along with X-ray imaging, in commercial systems (In-Vivo MS FX PRO, Bruker) and prototypes [11], although increasing the cost and complexity, including radiation protection.

The aim of this work is to explore a potential solution for generating adequately accurate synthetic morphological X-ray images by translating standard photographic images of mice using deep learning. Such an approach would benefit planar molecular imaging studies since the acquired photographic images have poor resolution in terms of anatomical information. By superimposing adequately high-resolution synthesized anatomical images on real functional mouse images, it would be possible to generate a preliminary estimation of the drug distribution within the body of the animal using conventional photographic sensors, thus reducing cost and complexity by eliminating the need for X-ray imaging of the animal.

For this purpose, we trained and tested a well-known conditional generative adversarial network for image-to-image translation (pix2pix cGAN) [12,13] in a dataset of 780 paired photographic/X-ray mice images. Conditional generative adversarial networks (cGANs) have been used previously for image synthesis in molecular imaging applications [14,15,16,17]. The preliminary results of the current study have been presented by our group elsewhere [18]. Compared to our previous study, we have properly balanced, extended and finalized the dataset in order to optimize the prediction in different potential inputs. Moreover, the achieved performance of the pix2pix network using different loss functions in the current dataset is presented. Finally, two commercial planar nuclear medicine preclinical scanners with a pre-installed photographic sensor have been used for further evaluation by superimposing the acquired 2D nuclear images with the corresponding trained network outputs in real-time during in vivo experiments.

## 2. Methodology

**Figure 1:**
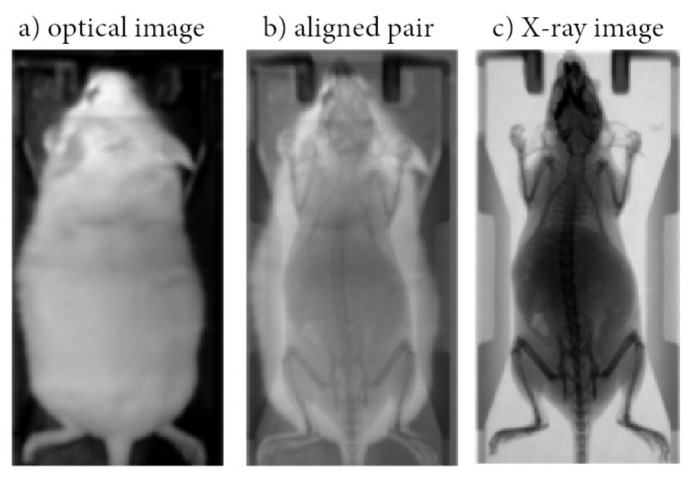
Indicative optical image acquired using a conventional photographic sensor located in BIOEMTECH eye’s series radioisotope screening tools (**a**); Indicative X-ray image acquired in a prototype PET/SPECT X-ray system (**c**) used as ground truth; Aligned pair (**b**).

A pix2pix cGAN was trained to map conventional photographic mouse images to X-ray scans [12]. Pix2pix is a common framework based on conditional Generative Adversarial Networks (cGANs), which predict pixels from pixels in any dataset, in which the aligned image pairs vary in the visual representation, but the renderings for, e.g., edges, stay the same (Figure 1). The above concept fits well to the presented dataset, as the field of view is clearly defined and the dimensions and weight of the mice usually involved in preclinical studies are similar. The methodology of the current work consists of 2 stages: (a) Data collection and preprocessing; (b) Modeling and performance evaluation.

### 2.1. Data Collection and Preprocessing

A dataset of 780 input/ground truth images has been acquired in order to train and test the pix2pix network. A photographic mouse image and the corresponding X-ray scan of the same anesthetized animal are referred to as input/ground truth images, respectively. The input images have been acquired using two commercial planar scanners for SPECT and PET radioisotope imaging, which contain a standard photographic sensor that provides an optical image of the animal as an anatomical mapping solution (eye’s series, BIOEMTECH, Greece) (Figure 1a). The X-ray images have been acquired with an X-ray tube (Source-Ray Inc, US) and a CMOS detector (C10900D, Hamamatsu, Japan), both mounted on a prototype PET/SPECT/X-ray system (Figure 1c) [11]. Both systems are adequate for mice imaging providing a useful field of view (FOV) of 50 mm × 100 mm.

We acquired 5 input/ground truth images of each animal in different poses upon the hosting bed. Given that small mice used in preclinical studies have similar dimensions and weight (∼20–40 g), we assume that the aforementioned methodology does not affect the training procedure and minimizes the number of laboratory animals used in the study. In all cases, animals were anesthetized with isoflurane and kept warmed during the scans. All animal procedures were approved by the General Directorate of Veterinary Services (Attica Prefecture, Athens, Greece) and the Bioethical Committee of the Institution (Permit number: EL 25 BIOexp 04).

**Figure 2:**
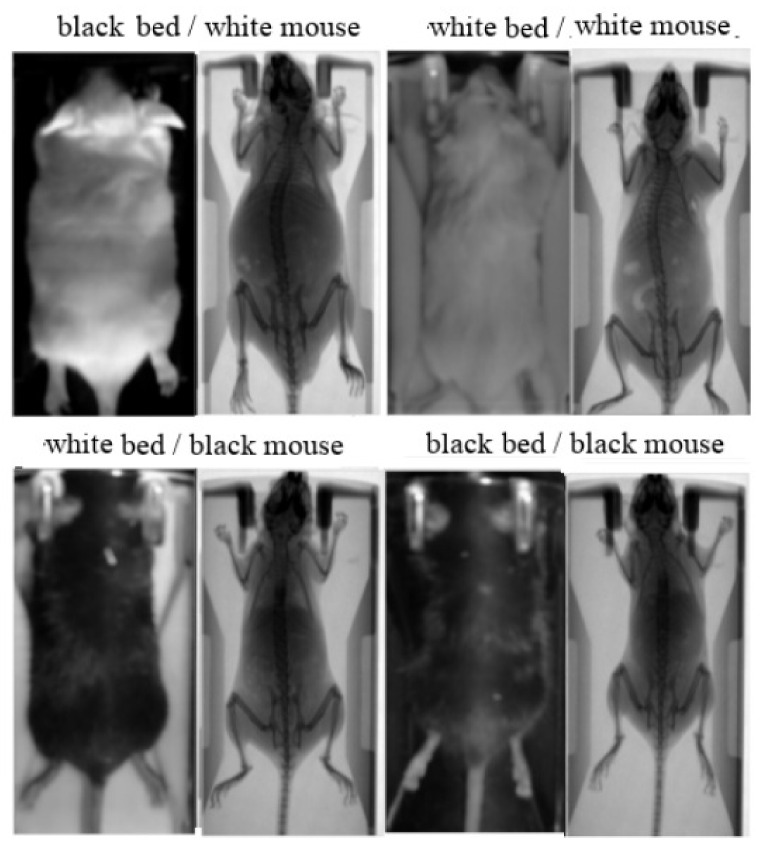
Aligned image pairs used for training. Photographic input image (**left**); Corresponding X-ray scan used as ground truth (**right**).

The study involved 78 white and 78 black Swiss albino mice, leading to a total number of 780 input/ground truth images. The mice were classified into two groups: (i) a group of 70 white and 70 black mice in order to collect the 700 paired images for training; (ii) a group of 8 white and 8 black mice in order to collect the 80 paired images for test and validation. Except mouse color, the method was evaluated against different animal hosting beds (plastic bed with white color; plastic bed with black color), which leads to different backgrounds in the input image. Four indicative input/ground truth pairs used for training and/or testing are illustrated in Figure 2. The paired images have been properly aligned prior to the training procedure, having 512 × 1024 pixels resolution, corresponding to the 50 mm × 100 mm field of view (Figure 1b). The detailed characteristics of the dataset are summarized in Table 1. The ratio between the test and train images was kept higher than 10% in each occasion, and the individual mice of the training set are separate from the individual mice of the test set.

### 2.2. Modeling and Performance Evaluation

Pix2pix is a cGAN (conditional Generative Adversarial Network) deep learning network that can be used for image-to-image translation. A cGAN is designed to create synthetic images *y* from a set of known images *x* and a randomly created noise vector *z* as follows:

(1) G:[x,z]→y

**Figure 3:**
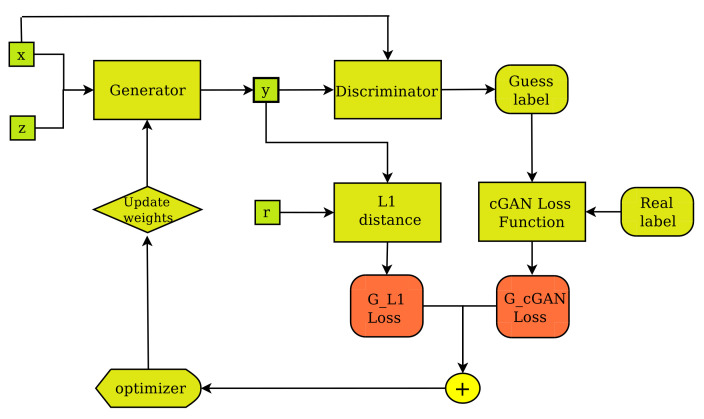
The pix2pix Generator’s training layout. The generator creates output image (y) from input image (x) and random noise vector (z) and improves its performance by receiving feedback from the discriminator, as well as regarding the degree of fakeness of the synthetic image (y) compared to the ground truth (r).

**Figure 4:**
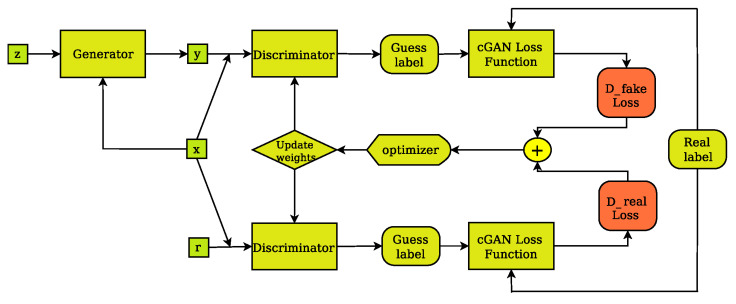
The cGAN discriminator’s training layout. The discriminator compares the input(x)/ground truth(r) pair of images and the input(x)/output(y) pair of images and outputs its guess about how realistic they look. The weights vector of the discriminator is then updated based on the classification error of the input/output pair (D fake Loss) and the input/target pair (D real Loss).

It consists of two main sub-networks, the generator (*G*) and the discriminator (*D*). The generator (Figure 3) is designed to create real-like, fake images, whereas the discriminator (Figure 4) is designed to classify images as real or fake. During training, the generator tries to improve its performance by updating its parameters based on input from the discriminator. This enables the generator to create better, more realistic fake images. The discriminator, on the other hand, improves its performance as a stand-alone network by learning to separate fake images from their real counterparts. The above ideas can be expressed mathematically in terms of a loss function as follows:

(2) $$LOSS_{cGAN}\left(G,D\right)=E_{x,y}\left[logD\left(x,y\right)\right]+E_{x,z}\left[log\left(1-D\left(x,G\left(x,z\right)\right)\right)\right]$$

The discriminator and the generator are adversaries in the sense that the generator attempts to minimize the Loss function, i.e., to fool the discriminator by producing real-like synthetic images, whereas the discriminator attempts to maximize the Loss function, i.e., expose imposter images. Training is complete when the generator is able to create fake, synthesized images, which are very difficult to be identified as fake by the discriminator. The pix2pix cGAN is further evolution of the above idea. Pix2pix is an implementation of the cGAN where the generation of an image is conditional on a specific target image instead of a set of target images. This way, a pix2pix generator (Figure 3) is not only trained to fool the discriminator but also to synthesize images that are as close as possible to the ground truth pair of each input image. This can be expressed mathematically by incorporating the idea of L1 distance between the paired generated image of the known input and the specific paired target image into Equation (2), as follows:

(3) $$LOSS_{pix2pix}=LOSS_{cGAN}\left(G,D\right)+\lambda \times L_{L1}\left(G\right)$$

where λ is a regularization parameter.

**Figure 5:**
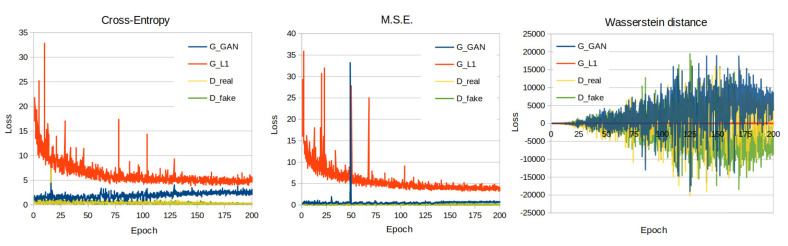
Training loss curves of the cross entropy, M.S.E. and Wasserstein distance loss function models.

In this study, we have utilized the PyTorch implementation [13] of the pix2pix algorithm as presented in [12]. In that implementation, the discriminator is designed as a PatchGAN, whereas the generator is designed as a U-Net. During the training process of all the models presented in this work, we used the default parameter and hyperparameter values of the PyTorch pix2pix implementation [13]. A list of values of important adjustable parameters for the training of the pix2pix models is illustrated in Table 2. In the present dataset, the cross entropy, which was used as the default cGan loss function in the pix2pix implementation of Isola et al. [12], was evaluated against the mean squared error (MSE) (squared L2 norm) loss used in LSGANs [19], the Wasserstein distance loss and wGANs [20]. The loss curves of the presented models’ training process are illustrated on Figure 5.

The performance evaluation of the pix2pix models in the present dataset has been performed using three image quality metrics: (a) peak signal-to-noise ratio (PSNR), (b) structural similarity index measure (SSIM) and (c) Fréchet inception distance (FID). PSNR is an expression for the ratio between the maximum possible signal power value and the power of distorting noise that affects the quality of its representation [21]; SSIM compares local patterns of pixel intensities that have been normalized for luminance and contrast [22]; FID is a metric focused on assessing the quality of images created by generative models and is considered one of the most widely accepted image quality metrics for image generation [23,24]. FID compares the distribution of the generated images with the distribution of the set of the target images and captures the similarity between the two sets. The aforementioned metrics have been used previously to assess the quality of cGAN-generated images quantitatively in comparison to other metrics, such as mean absolute error (MAE) and mean square error (MSE), which, in some cases, are not appropriate for evaluating the results of the cGAN approach [25,26,27,28].

## 3. Results

### 3.1. Quantitative Evaluation

**Figure 6:**
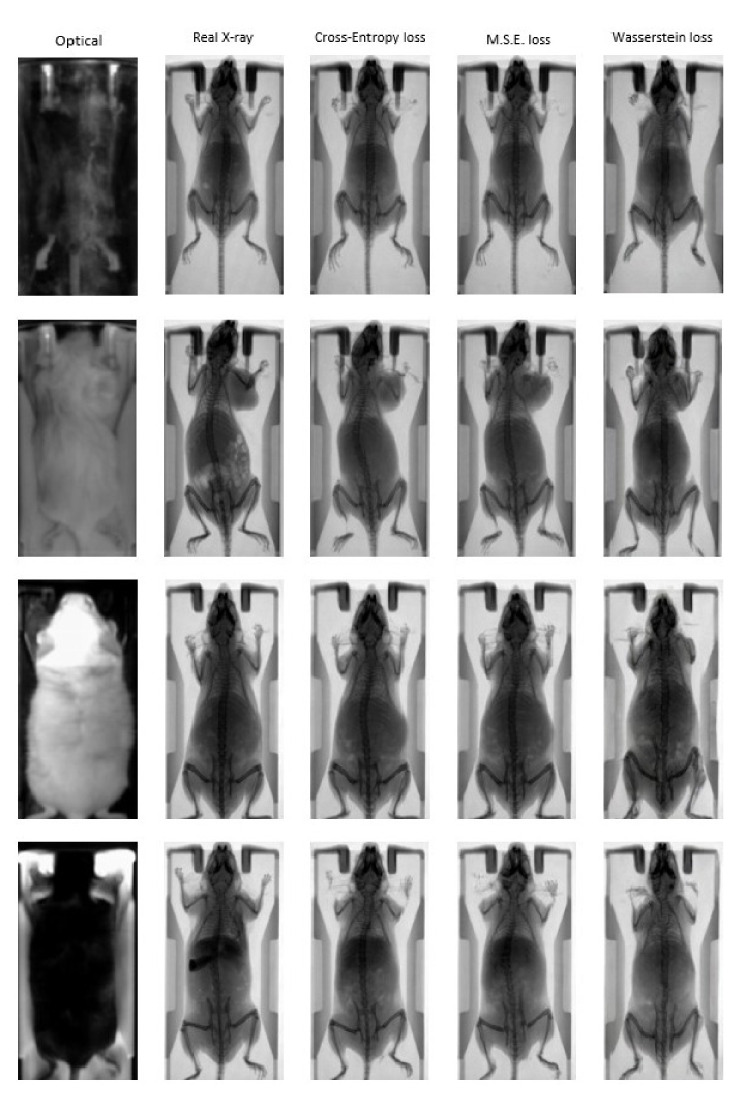
Indicative “fake” X-ray images from the pix2pix trained network using different loss functions: Cross Entropy (3rd column); MSE (4th column); Wasserstein distance (5th column). The input photographic images and the corresponding ground truth images are presented in the first two columns.

Figure 6 illustrates indicative optical to X-ray translations in the test dataset for the four distinct combinations of mouse and bed color that the pix2pix network has been trained on. The input optical image and the ground truth are presented alongside synthetic X-ray images generated by the pix2pix network that was trained using three different loss functions: (a) Cross Entropy; (b) MSE; (c) Wasserstein distance. The results demonstrate the ability of the trained networks to efficiently map the photographic mouse image to an X-ray scan in terms of animal mapping, but not for diagnosis, as the information of the actual structure of mouse cannot be generated. However, the renderings and the morphological characteristics are reproduced in an adequate manner compared to the ground truth X-ray images, demonstrating the feasibility of the proposed approach for animal mapping.

Quantitative evaluation of network outputs compared to ground truth images has been performed using three performance metrics: (a) PSNR; (b) SSIM; (c) FID in the test dataset. According to the calculated values presented in Table 3, the pix2pix network trained using Cross Entropy as the cGan loss function is able to generate X-ray images that are more accurate translations of the photographic image to the real X-ray scan compared to MSE and/or Wasserstein distance loss functions.

Table 4 presents the calculated metrics of the pix2pix Cross Entropy model on the different combinations of mouse and bed color. According to the FID values, the photographic images that have different colors of bed than the colors of the mouse are translated into images that resemble the ground truth better. This can be explained by (a) the greater percentage of such cases within the dataset, meaning the network is better trained on such cases, and (b) the greater contrast of the mouse against the background, which makes it easier for the network to distinguish the different subjects.

### 3.2. Animal Mapping during In Vivo Molecular Imaging Experiments

The proposed trained network was used for anatomical mouse mapping in two, proof of concept, nuclear molecular imaging experiments. Two planar preclinical scanners for SPECT and PET isotopes imaging have been used (eye’s series, BIOEMTECH, Greece). The scanners come with a pre-installed photographic sensor, which is used to superimpose standard photography of the mouse with the acquired nuclear image for anatomical mapping purposes. The trained with the Cross Entropy loss function pix2pix network was used to generate an X-ray scan from the acquired photographic image in real-time during in vivo experiments. The output was then superimposed in real-time with the corresponding functional (PET or SPECT) image to provide high-resolution information for anatomical mapping.

**Figure 7:**
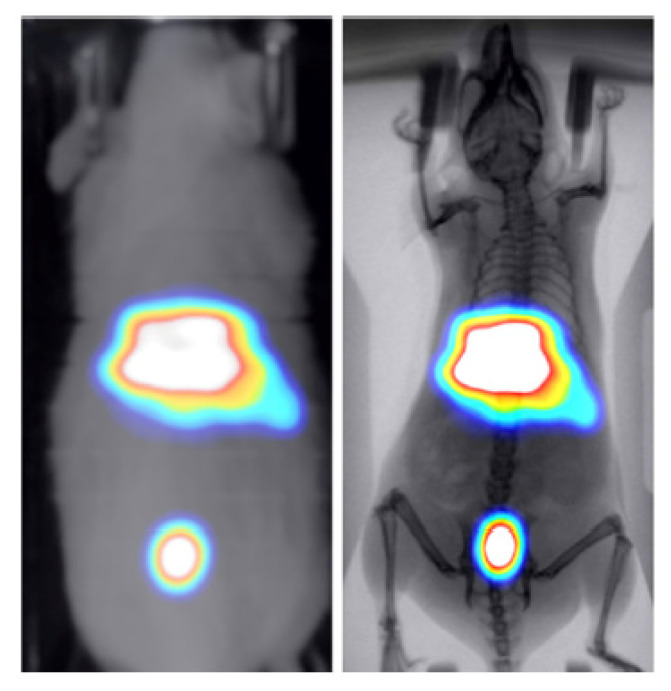
${}^{99m}Tc$-MDP-labelled nuclear image of a healthy mouse fused with the optical image provided in the γ-eye scintigraphic system (**left**) and the X-ray produced from the pix2pix trained network (**right**).

**Figure 8:**
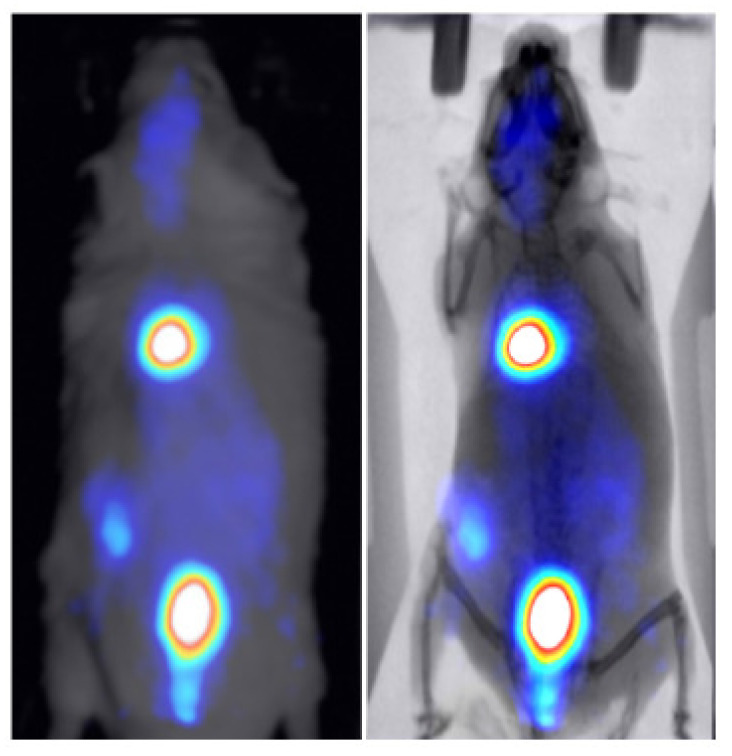
${}^{18}F$-FDG nuclear image of a healthy mouse fused with the optical image provided in the β-eye planar coincidence imaging system (**left**) and the X-ray produced from the pix2pix trained network (**right**).

Two healthy mice were administered through tail vein injection with 286 uCi/50 uL of technetium-99m (${}^{99m}Tc$) and 30 uCi/100 uL of 2-deoxy-2-[${}^{18}F$]fluoro-D-glucose ([${}^{18}F$] FDG) to study the kinetics of these widely used tracers in BIOEMTECH’s γ-eye scintigraphic system and β-eye planar coincidence imaging system, respectively. Figure 7 shows the ${}^{99m}Tc$ nuclear image fused with the optical one provided in the γ-eye system and with the predicted X-ray image produced from the pix2pix network. The nuclear image shows the clear targeting of the tracer and the biodistribution in the liver, spleen and bladder, as the main organs of accumulation. Figure 8 shows the [${}^{18}F$]FDG nuclear image fused with the optical one provided in the β-eye system and with the predicted X-ray image produced from the pix2pix network. The nuclear image shows the accumulation of the compound in the brain, heart, liver, intestines and bladder, as expected. In both cases, the produced X-ray superimposed with the corresponding nuclear image provides an anatomical map of the small animal that enhances the overall image information.

## 4. Discussion and Conclusions

Image-to-image translation techniques have been used in medical imaging for several tasks, including segmentation, denoising, super-resolution, modality conversion and reconstruction [27]. In the current work, we present an off-the-shelf approach for generating adequately accurate synthetic morphological X-ray images by translating standard photographic mice images. Artificially produced X-ray mouse images can be superimposed with functional radioisotope or fluorescence/bioluminescence 2D images to enhance overall anatomical information. Although not suitable for diagnosis, such information can be used for animal mapping in preclinical planar molecular imaging systems without adding the costs and complexity of radiation sources, radiation protection and animal irradiation during repeated scans.

A well-known cGAN network (pix2pix) that learns a mapping from the input image to the output image has been trained in a 700 input (mouse photography)/ground truth (mouse X-ray scan) dataset and evaluated in a 80 input/ground truth dataset. The pix2pix network has been evaluated against three different loss functions: (a) Cross Entropy, (b) MSE and (c) Wasserstein distance, with the first two achieving similar performance superior to the Wasserstein distance. However, the results in all cases show that the network predicts an X-ray image with sufficient accuracy and that the calculated metrics are comparable with those presented in other studies that have evaluated pix2pix networks. In [28], four approaches including the pix2pix network, which was used as a baseline, were evaluated on a well-known dataset primarily presented in [29]. The SSIM and PSNR values of the pix2pix model on that dataset were calculated equal to 0.286 and 12.868, respectively. In [30], a pix2pix network was used as a baseline and was evaluated on datasets presented in [31,32]. The SSIM and FID values of the pix2pix model on the [31] dataset were calculated equal to 0.770 and 66.752, respectively, while FID values on three datasets presented in [32] were 96.31 (handbag dataset), 197.492 (shoes dataset) and 190.161 (clothes dataset). In [33], four pix2pix variations were evaluated on two datasets presented in the same study and the original pix2pix network was used as a baseline. The FID scores of pix2pix on MR-to-CT scan and CT-to-MR scan translation tasks were 122.6 and 90.8, respectively. Although different problems are presented in the aforementioned studies, achieved metrics (Table 3) are indicative of the success of the approach in our dataset.

Two proof-of-concept nuclear molecular imaging in vivo experiments have been conducted in order to demonstrate the efficacy of the method in terms of enhancing anatomical information in functional imaging preclinical studies. For that purpose, two commercial planar radioisotope imaging systems with a pre-installed conventional photographic sensor have been used (eye’s series, BIOEMTECH, Greece). The produced X-ray superimposed with the corresponding nuclear image provides an accurate morphological map of the small animal that is used to better identify the organs that the studied compound has accumulated.

Future work is oriented in expanding our dataset with data augmentation techniques in order to improve the generalization of our method and hyperparameter tuning in order to further tailor the behavior of the pix2pix network to the final dataset. Taking into account that the primary information comes from photographic sensors and given that small mice used in preclinical studies have similar dimensions and weight (∼20–40 g), geometric and/or photometric transformations may expand the dataset without losing the generalization of the approach, thus providing better performance metrics. 

## Figures and Tables

**Table 1:** Train and test dataset detailed characteristics.

Mouse Color	Bed Color	Train	Test	Test/Train (%)
White	Black	260	30	11.5
White	White	90	10	11.1
Black	White	260	30	11.5
Black	Black	90	10	11.1
	**Total**	**700**	**80**	

**Table 2:** Values of important adjustable training parameters and hyperparameters.

Parameter	Value
Learning rate	0.0002
Beta 1 parameter for the optimizer (adam)	0.5
Beta 2 parameter for the optimizer (adam)	0.999
Maximum epochs	200
Lambda (λ) weight for the L1 loss	100
Generator layers	8
Discriminator layers	3
Load size	512
Mini batch size	1

**Table 3:** Metrics of the different cGAN loss functions tested.

cGAN Loss Function	PSNR ↑	SSIM ↑	FID ↓
Cross entropy	21.923	0.771	85.428
MSE	21.954	0.770	90.824
Wasserstein distance	17.952	0.682	162.015

**Table 4:** Metrics of the pix2pix Cross Entropy model on the different combinations of mouse and bed color.

Mouse Color	Bed Color	PSNR ↑	SSIM ↑	FID ↓
black	white	22.808	0.791	112.948
black	black	22.894	0.794	151.006
white	black	21.196	0.750	109.116
white	white	20.270	0.743	163.056

## Data Availability

Not applicable.

## References

[1] Willmann J., van Bruggen N., Dinkelborg L., Gambhir S. (2008). Molecular imaging in drug development. Nat. Rev. Drug Discov..

[2] Pysz M.A., Gambhir S.S., Willmann J.K. (2010). Molecular imaging: Current status and emerging strategies. Clin. Radiol..

[3] Kagadis G., Ford N., Karbanatidis D., Loudos G. (2016). Handbook of Small Animal Imaging.

[4] Paul S.M., Mytelka D.S., Dunwiddie C.T., Persinger C.C., Munos B.H., Lindborg S.R. (2010). How to improve RD productivity: The pharmaceutical industry’s grand challenge. Nat. Rev. Drug Discov..

[5] US Food and Drug Administration (2015). US Department of Health and Human Services.

[6] Mannheim J.G., Kara F., Doorduin J., Fuchs K., Reischl G., Liang S., Verhoye M., Gremse F., Mezzanotte L., Huisman M.C. (2018). Standardization of Small Animal Imaging-Current Status and Future Prospects. Mol. Imaging Biol..

[7] Stuker F., Ripoll J., Rudin M. (2011). Fluorescence Molecular Tomography: Principles and Potential for Pharmaceutical Research. Pharmaceutics.

[8] Debie P., Lafont C., Defrise M., Hansen I., van Willigen D.M., van Leeuwen F., Gijsbers R., D’Huyvetter M., Devoogdt N., Lahoutte T. (2020). Size and affinity kinetics of nanobodies influence targeting and penetration of solid tumours. J. Control Release.

[9] Georgiou M., Fysikopoulos E., Mikropoulos K., Fragogeorgi E., Loudos G. (2017). Characterization of g-eye: A low cost benchtop mouse sized gamma camera for dynamic and static imaging studies. Mol. Imaging Biol..

[10] Zhang H., Bao Q., Vu N., Silverman R., Taschereau R., Berry-Pusey B., Douraghy A., Rannou F., Stout D., Chatziioannou A. (2011). Performance evaluation of PETbox: A low cost bench top preclinical PET scanner. Mol. Imaging Biol..

[11] Rouchota M., Georgiou M., Fysikopoulos E., Fragogeorgi E., Mikropoulos K., Papadimitroulas P., Kagadis G., Loudos G. (2017). A prototype PET/SPET/X-rays scanner dedicated for whole body small animal studies. Hell. J. Nucl. Med..

[12] Isola P., Zhu J.-Y., Zhou T., Efros A. Image-to-image translation with conditional adversarial networks. Proceedings of the IEEE Conference on Computer Vision and Pattern Recognition Proceedings.

[13] Paszke A., Gross S., Massa F., Lerer A., Bradbury J., Chanan G., Killeen T., Lin Z., Gimelshein N., Antiga L. PyTorch: An Imperative Style, High-Performance Deep Learning Library. Proceedings of the Advances in Neural Information Processing Systems.

[14] Gong K., Yang J., Larson P.E.Z., Behr S.C., Hope T.A., Seo Y., Li Q. (2021). MR-based Attenuation Correction for Brain PET Using 3D Cycle-Consistent Adversarial Network. IEEE Trans. Radiat. Plasma Med. Sci..

[15] Amyar A., Ruan S., Vera P., Decazes P., Modzelewski R. RADIOGAN: Deep Convolutional Conditional Generative Adversarial Network to Generate PET Images. Proceedings of the 7th International Conference on Bioinformatics Research and Applications (ICBRA).

[16] Denck J., Guehring J., Maier A., Rothgang E. (2021). Enhanced Magnetic Resonance Image Synthesis with Contrast-Aware Generative Adversarial Networks. J. Imaging.

[17] Ouyang J., Chen K.T., Gong E., Pauly J., Zaharchuk G. (2019). Ultra-low-dose PET reconstruction using generative adversarial network with feature matching and task-specific perceptual loss. Med. Phys..

[18] Fysikopoulos E., Rouchota M., Eleftheriadis V., Gatsiou C.-A., Pilatis I., Sarpaki S., Loudos G., Kostopoulos S., Glotsos D. Photograph to X-ray Image Translation for Anatomical Mouse Mapping in Preclinical Nuclear Molecular Imaging. Proceedings of the 2021 International Conference on Medical Imaging and Computer-Aided Diagnosis (MICAD 2021).

[19] Mao X., Li Q., Xie H., Lau R.Y.K., Wang Z., Smolley S.P. Least Squares Generative Adversarial Networks. Proceedings of the 2017 IEEE International Conference on Computer Vision (ICCV).

[20] Arjovsky M., Chintala S., Bottou L. (2017). Wasserstein GAN. arXiv.

[21] Sara U., Akter M., Uddin M. (2019). Image Quality Assessment through FSIM, SSIM, MSE and PSNR—A Comparative Study. J. Comput. Commun..

[22] Wang Z., Bovik A.C., Sheikh H.R., Simoncelli E.P. (2004). Image quality assessment: From error visibility to structural similarity. IEEE Trans. Image Process..

[23] Heusel M., Ramsauer H., Unterthiner T., Nessler B., Hochreiter S. GANs Trained by a Two Time-Scale Update Rule Converge to a Local Nash Equilibrium. Proceedings of the Advances in Neural Information Processing Systems.

[24] Benny Y., Galanti T., Benaim S., Wolf L. (2021). Evaluation Metrics for Conditional Image Generation. Int. J. Comput. Vis..

[25] Salimans T., Goodfellow I., Zaremba W., Cheung V., Radford A., Chen X. Improved techniques for training gans. Proceedings of the Advances in Neural Information Processing Systems.

[26] Kupyn O., Budzan V., Mykhailych M., Mishkin D., Matas J., DeblurGAN J. Blind motion deblurring using conditional adversarial networks. Proceedings of the IEEE Conference on Computer Vision and Pattern Recognition.

[27] Kaji S., Kida S. (2019). Overview of image-to-image translation by use of deep neural networks: Denoising, super-resolution, modality conversion, and reconstruction in medical imaging. Radiol. Phys. Technol..

[28] Yoo J., Eom H., Choi Y. (2019). Image-to-image translation using a crossdomain auto-encoder and decoder. Appl. Sci..

[29] Cordts M., Omran M., Ramos S., Rehfeld T., Enzweiler M., Benenson R., Franke U., Roth S., Schiele B. The cityscapes dataset for semantic urban scene understanding. Proceedings of the IEEE Conference on Computer Vision and Pattern Recognition.

[30] Albahar B., Huang J. Guided Image-to-Image Translation With Bi-Directional Feature Transformation. Proceedings of the IEEE/CVF International Conference on Computer Vision (ICCV).

[31] Liu Z., Luo P., Qiu S., Wang X., Tang X. DeepFashion: Powering Robust Clothes Recognition and Retrieval with Rich Annotations. Proceedings of the IEEE Conference on Computer Vision and Pattern Recognition (CVPR).

[32] Xian W., Sangkloy P., Agrawal V., Raj A., Lu J., Fang C., Yu F., Hays J. TextureGAN: Controlling Deep Image Synthesis with Texture Patches. Proceedings of the IEEE Conference on Computer Vision and Pattern Recognition (CVPR).

[33] Paavilainen P., Akram S.U., Kannala J. Bridging the Gap Between Paired and Unpaired Medical Image Translation. Proceedings of the MICCAI Workshop on Deep Generative Models.

